# Cognitive function in patients with neuroborreliosis: A prospective cohort study from the acute phase to 12 months post treatment

**DOI:** 10.1002/brb3.2608

**Published:** 2022-05-20

**Authors:** Silje Andreassen, Anne Marit Solheim, Unn Ljøstad, Åse Mygland, Åslaug Rudjord Lorentzen, Harald Reiso, Mona Kristiansen Beyer, Hanne Flinstad Harbo, Gro Christine Christensen Løhaugen, Randi Eikeland

**Affiliations:** ^1^ Department of Pediatrics Sørlandet Hospital Arendal Norway; ^2^ Institute of Clinical Medicine University of Oslo Oslo Norway; ^3^ Department of Neurology Sørlandet Hospital Kristiansand Norway; ^4^ Department of Clinical Medicine University of Bergen, Bergen Norway; ^5^ Department of Habilitation Sørlandet Hospital Kristiansand Norway; ^6^ The Norwegian National Advisory Unit on Tick‐borne diseases Kristiansand Norway; ^7^ Division of Radiology and Nuclear Medicine, Oslo University Hospital Oslo Norway; ^8^ Department of Neurology Oslo University Hospital Oslo Norway; ^9^ Department of Health and Nursing Sciences University of Agder Kristiansand Norway

**Keywords:** cognitive, fatigue, Lyme, neuroborreliosis, prognosis

## Abstract

**Background:**

Long‐term cognitive problems after neuroborreliosis treatment remain a subject of debate. We have previously shown that cognitive problems are not present in the acute phase of neuroborreliosis, although fatigue is common. The aim of this study was to re‐assess the same patient cohort and evaluate long‐term outcomes.

**Methods:**

In this follow‐up, we re‐assessed 58 patients with well‐characterized neuroborreliosis 12 months after completing treatment. The same protocol with eight subtests measuring attention and processing speed and the Fatigue Severity Scale (FSS) were used to compare the results from the acute phase to 12 months post treatment.

**Results:**

We found no changes in attention or processing speed but a reduction in the level of fatigue (median score on FSS: 4.9 vs. 3.9, *p *< .001) from the acute phase to 12 months post treatment.

**Conclusion:**

The patient group did not develop problems with attention or processing speed post treatment, while the level of fatigue decreased.

## Abstract 2

In this follow‐up we re‐assessed patients with neuroborreliosis 12 months post treatment. There were no changes in the cognitive functions beeing assessed, but level of fatigue decreased. Our findings suggest a favourble outcome regarding cogntive function after well treated neuroborreliosis.

## 1 INTRODUCTION

Neuroborreliosis is a vector‐borne infection in the nervous system caused by the bacterium *Borrelia burgdorferi*. It is typically characterized by lymphocytic meningoradiculitis affecting the cranial nerves and/or the peripheral spinal nerve roots (Mygland et al., 2010). Persisting symptoms such as cognitive problems and fatigue more than 6 months after a confirmed and adequately treated borrelia infection are referred to as post‐Lyme disease syndrome (PLDS; Wormser et al., 2006). PLDS is a highly debated phenomenon, and both its prevalence and characteristics are not clearly understood (Eikeland et al., 2012; Keilp et al., 2018). Some studies describe attention deficits, reduced processing speed and fatigue in patients treated for borreliosis (Chandra et al., 2013; Eikeland et al., 2016, 2020; Keilp et al., 2006, 2018; Pollina et al., 1999). However, a review from Dersch et al. and a large register‐based study from Denmark do not support these findings (Dersch et al., 2016; Obel et al., 2018).

Our study group (BorrSci, 2021) recently published data on processing speed, attention, and fatigue in patients with acute neuroborreliosis. We did not find any differences in attention or processing speed, but found a higher level of fatigue in patients, compared to a matched control group (Andreassen et al., 2021). Whether reduced cognitive function develops after treatment, and fatigue decreases with time, are the research questions we hope to enlighten in this study. In this study, the same cohort of patients with neuroborreliosis was tested with the same assessment protocol 12 months after treatment.

## 2 MATERIALS AND METHODS

### 2.1 Recruitment and participants

This study is part of a Norwegian multicenter treatment and prognosis trial comparing 2 and 6 weeks of doxycycline treatment for neuroborreliosis (BorrSci, 2021; Solheim et al., 2019). Patients aged ≥18 years with possible or definite neuroborreliosis according to the European Federation of Neurological Societies criteria (Mygland et al., 2010) were included. Between November 2015 and December 2018, we recruited patients with acute neuroborreliosis and assessed attention, processing speed and fatigue within 4 weeks after treatment started. A healthy control group matched on gender and age was included for comparison in the acute phase, but was not retested at 12 months. Descriptions of the subjects, inclusion/exclusion criteria and results have been published previously (Andreassen et al., 2021). Twelve months after completing antibiotic treatment, we re‐assessed the patient group with the same neuropsychological tests and fatigue questionnaire as in the acute phase. Demographic and clinical data for the 58 patients were collected at inclusion, and data on working status were collected 12 months post treatment.

### 2.2 Neuropsychological tests

Attention was assessed by using the Wechsler Intelligence Scale, fourth edition. Digit span forward/backward (Wechsler, 1997b), Wechsler Memory Scale, third edition, spatial span (Wechsler, 1997a) and color–word interference test from Delis–Kaplan Executive Function System (D‐KEFS; Delis et al., 2001a). Processing speed was measured with trail making test motor speed and color words read from the D‐KEFS. The analysis is based on raw scores. The raw score on the color–word read, inhibition, and trail making test is the time to complete the task measured in seconds. On digit span and spatial span, the patient is given one point for each correct sequence, and raw scores refer to the number of points in total on each subtest. All test batteries are standardized, validated, translated to Norwegian, and frequently used in clinical practice.

### 2.3 Other variables

We used the Fatigue Severity Scale (FSS; Krupp et al., 1989) to assess the level of fatigue. The FSS is a 9‐item scale and has been translated and validated in the Norwegian population. A score ≥5 is considered severe (Lerdal et al., 2005).

We used the level of education and occupational status based on the Hollingshead index to estimate the level of socioeconomic status (SES). The Hollingshead index ranges from 1 to 5, where 5 is the highest level of SES (Hollingshead, 2005, 2017).

At the 12‐month follow‐up, we asked patients about their working status. Patients on sick leave were asked if their absence was due to neuroborreliosis.

### 2.4 Statistics

The statistical software SPSS Statistics 25 was used for all analyses. We used a paired sample *t*‐test to compare the mean difference in raw scores between the acute phase and 12 months post treatment. The Wilcoxon signed‐rank test and Mann–Whitney *U*‐test were chosen if data did not meet the criteria for parametric analysis. We used Spearman's rho to correlate FSS with neuropsychological tests, as data from FSS were not normally distributed. We dichotomized the computed FSS scores into two categories, ≥5 or < 5, and used McNemar tests to decide if the proportion of severe fatigue changed significantly from the acute phase until 12 months post treatment. Independent samples *t‐*tests and Mann–Whitney *U*‐tests were used to identify possible differences in neuropsychological test scores and FSS scores between patients with definite and possible neuroborreliosis 12 months post treatment.

Bonferroni was used to adjust for multiple comparisons; the adjusted *p*‐value was .006.

### 2.5 Ethics

The study is part of the BorrSci project (Lyme borreliosis; a scientific approach to reduce diagnostic and therapeutic uncertainties) and is approved by the Norwegian Regional Committee for Medical and Health Research Ethics, the southeastern region (2015/1031 and 2015/1588) as well as through local routines at Sørlandet Hospital and Oslo University Hospital. All participants gave written informed consent.

## 3 RESULTS

Out of 72 patients included and tested in the acute phase of neuroborreliosis, 14 patients, nine male and five female, were lost to follow‐up. In total, 58 patients were tested both in the acute phase and 12 months after treatment. Patient characteristics are presented in Table 1. Of the patients lost to follow‐up, seven declined to participate, three did not respond to call, three declined due to illness not related to neuroborreliosis, and one patient moved to another part of the country. Eleven patients were classified as definite neuroborreliosis, while three were classified as possible. Mean age and SES did not differ from the cohort and were 54.4 (range 20–78) and 3.1, respectively. There were no differences between patients lost to follow‐up and the cohort in neuropsychological test scores or FSS collected in the acute phase. Patients with possible neuroborreliosis had neurological symptoms indicating neuroborreliosis, specifically meningoradiculitis (76.9%) and/or cranial nerve affection (38.5%). All of them had leukocytosis in the cerebrospinal fluid (> 5 cells) but no intrathecal antibody production. We found no differences between patients with possible or definite neuroborreliosis in either neuropsychological test scores or FSS 12 months post treatment.

**TABLE 1:** Demographic and clinical data

Acute phase (*n *= 58)	
Mean age (range)	58 (30–81)
Gender (%)	
Male	27 (46.6)
Female	31 (53.4)
Mean socioeconomic status	3.4
Possible neuroborreliosis (%)	11 (19.0)
Definite neuroborreliosis (%)	47 (81.0)

**12 months follow‐up (*n* = 58)**	
Working status (%)	
Full‐time work	21 (36.2)
Part‐time work	2 (3.4)
Age pension	23 (39.7)
Disability pension	5 (8.6)
Full‐time sick	3 (5.2) 1 due to neuroborreliosis
Part‐time sick	3 (5.2) 2 due to neuroborreliosis
Unemployed	1 (1.7)

We found no differences in mean scores on neuropsychological tests between the acute phase and 12 months after treatment (see Table 2). Missing data did occur; two patients missed spatial span, one patient was not able to perform color–word reading, color–word inhibition, and trail making motor, and one patient missed color–word inhibition due to colorblindness. Three patients did not fill out FSS. After correcting for multiple testing, there were no differences in neuropsychological test scores when comparing results within the group with definite neuroborreliosis only (*n* = 47).

**TABLE 2:** Attention and processing speed, mean difference in raw scores from the acute phase to the 12‐month follow‐up

	Acute phase	Follow‐up	Mean difference (95% CI)	*t*‐value	*df*	*p*‐value
Digit span forward	8.6 (2.0)	8.7 (2.3)	−0.1 (−0.560–0.456)	−0.204	57	.839
Digit span backward	7.6 (1.9)	7.9 (2.3)	−0.3 (−0.702–0.116)	−1.435	57	.157
Spatial span forward	7.3 (1.8)	7.4 (1.7)	−0.2 (−0.593–0.272)	−0.744	55	.460
Spatial span backward	6.7 (2.0)	7.1 (1.9)	−0.4 (−0.944–0.051)	−1.798	55	.078
Spatial span total	13.9 (3.2)	14.5 (3.1)	−0.5 (−1.254–0.219)	−1.409	55	.164
CW read s	22.3 (6.2)	22.9 (4.9)	−0.6 (−2.140–0.912)	−0.806	56	.424
CW inhibition s	60.3 (17.0)	60.0 (19.0)	0.3 (−2.621–3.264)	0.219	55	.828
TMT motor s	26.6 (11.7)	25.0 (9.5)	1.6 (−0.712–3.835)	1.376	56	.174

Abbreviations: CW, color word;; TMT, Trail Making Test.

* Level of significance after Bonferroni correction is *p* < .006.

The level of fatigue decreased from the acute phase until follow‐up. Both the median score on FSS and the proportion of patients with severe fatigue ≥5 decreased (see Table 3). There was no correlation between fatigue and attention or processing speed.

**TABLE 3:** Level of fatigue acute phase and 12‐month follow‐up, all patients, and patients with definite neuroborreliosis

	All patients			Definite neuroborreliosis		
	Acute phase *n* = 58	Follow‐up *n* = 55	*p*‐value	Acute phase *n* = 47	Follow‐up *n* = 44	*p*‐value
Median FSS	4.9	3.9	<.001*	5.3	3.8	.002*
FSS < 5 or ≥5			.001*			.008
FSS < 5 (%)	28 (50.9)	43 (78.2)		21 (47.7)	33 (75.0)	
FSS ≥5	27 (49.1)	12 (21.8)		23 (52.3)	11 (25.0)	

Abbreviations: FSS, Fatigue Severity Scale.

* Level of significance after Bonferroni correction is *p* < .006.

## 4 DISCUSSION

We found no reduction in attention or processing speed between the acute phase and the 12‐month follow‐up. The level of fatigue decreased (median scores 4.9 vs. 3.9), but 20% of the patients still reported severe fatigue 12 months after treatment. These numbers correspond well to the level of fatigue in the Norwegian population (Lerdal et al., 2005). Ten percent of the patients were on sick leave, of which half of them related this to health problems after neuroborreliosis. Regarding cognitive function, our findings suggest a positive long‐term outcome for patients with neuroborreliosis. The results are in line with other studies, which include an assessment of cognitive function after treated neuroborreliosis (Dersch et al., 2015; Kaplan et al., 2003). Dersch et al. included 30 patients with definite neuroborreliosis and examined them with the Mini‐Mental State Exam and a verbal list‐learning task in addition to the FSS 4 years after treatment. They found no difference in cognitive function or level of fatigue, compared to a healthy control group. However, the Mini‐Mental State Exam might have been insufficient to identify subtle cognitive changes in patients with neuroborreliosis, as it is a screening tool primarily used to identify cognitive impairment in elderly individuals. Kaplan et al. assessed 125 patients with a neuropsychological test battery and compared the results with age‐reference normative data. Attention, executive functions, and memory were all within the normal range, while processing speed was not investigated. However, the inclusion criteria were different from ours, as they included patients with a history of borreliosis who were both seronegative and seropositive for IgG antibodies against *B. burgdorferi*. Furthermore, all the patients had residual symptoms, including self‐reported cognitive problems at inclusion.

In infectious diseases other than neuroborreliosis, both attention and processing speed are cognitive domains that appear to be especially vulnerable (Fisher & Bernard, 2019; Meng et al., 2017). As we assumed, patients with neuroborreliosis might be more susceptible to a decrease in these functions as well, we included tests that assessed both attention and processing speed. Some studies have included neuropsychological tests measuring processing speed and attention with slightly diverging results. In a study on 25 patients with a history of disseminated borreliosis, Pollina et al. found deficits in initiating speed, but not generalized slowing, on cognitive demanding tasks, compared with a healthy control group (Pollina et al., 1999). In a study of 50 patients with neuroborreliosis 30 months after treatment, Eikeland et al. (2012) found a reduction in both attention and processing speed on some, but not all, tasks, compared to a control group. Touradji et al. (2018) found a reduction in verbal memory but also reduced processing speed in a subgroup of PLDS patients, where over 90% of patients reported subjective cognitive problems at inclusion. Only a quarter of these patients showed cognitive deficits on objective neuropsychological tests.

These studies differ from ours, as the patients were included long after treatment started and were not re‐assessed with the same protocol twice to look at changes. In both the study by Pollina et al. and Touradij et al., cognitive problems and/or fatigue were one of the inclusion criteria, making them selected groups in that respect. However, our sample, who were included from the acute phase of neuroborreliosis, did not have a history of persistent cognitive problems or fatigue. We found no cognitive deficits in patients with acute neuroborreliosis but still wanted to see if cognitive problems could develop in the post‐infectious phase. However, our results do not support this.

Nevertheless, we cannot reject that domains other than attention and processing speed might be affected. There are several reports on reduced verbal learning and memory in patients with neuroborreliosis after treatment (Eikeland et al., 2012; Keilp et al., 2006; Westervelt & McCaffrey, 2002). Research on other cognitive domains, such as executive functions, has presented conflicting results. While some find lower scores on certain executive tests in patients with neuroborreliosis (Eikeland et al., 2012; Benke et al., 1995; Schmidt et al., 2015), others find no differences at all (Kaplan et al., 1999, 2003). Both various inclusion criteria and differences in the populations might explain the conflicting results. Different choices of tests could also influence the results. Executive functions are a cluster of functions including but not limited to flexibility, inhibition of impulses, problem‐solving and initiating. One test will not capture the whole cluster of executive functions.

Level of fatigue was the only factor that changed significantly during the follow‐up. Although the patients reported a higher level of fatigue 12 months post treatment, compared to the healthy control group 1 year earlier, 3.9 versus 2.9 (Andreassen et al., 2021), their fatigue score 12 months post treatment corresponds with the normative level of fatigue in the Norwegian population. Lerdal et al. found the mean level of fatigue measured with FSS to be 3.9, and 20% of Norwegians had high or severe fatigue, defined as a score ≥5 (Lerdal et al., 2005). Our results regarding fatigue are in line with other studies where fatigue decreases and the long‐term prognosis after neuroborreliosis is favorable (Eikeland et al., 2020; Obel et al., 2018).

The strength of our study is a well‐defined neuroborreliosis patient group, followed from the acute phase to look at possible long‐term effects on attention, processing speed and fatigue.

The main weakness is that 14 patients were lost to follow‐up. Even though the patients lost to follow‐up did not have higher fatigue or lower scores on neuropsychological tests in the acute phase, this has lowered the population size during the 12‐month follow‐up. Furthermore, a more comprehensive test protocol assessing other domains could have yielded different results. We included a healthy control group in the acute phase, but they were not retested in this follow‐up, as we did not expect the control group to change their cognitive function or level of fatigue in one year. There is always a possibility for practice effects when retesting; however, different neuropsychological tests are prone to practice effects to varying degrees. Tests that rely on a novelty effect or measure memory are more susceptible to practice effects (Strauss et al., 2006). In this study, we used neuropsychological tests that have a low to moderate practice effect (Delis et al., 2001c; Psychological Corporation, 1997; Wechsler, 2008b; Strauss et al., 2006). Nevertheless, we cannot completely rule out the possibility of some practice effects in the patient group.

## 5 CONCLUSION

The outcome after adequately treated neuroborreliosis seems favorable, and our findings suggest no reduction in attention or processing speed. The level of fatigue decreased significantly during the follow‐up. Twenty percent of the patients still reported severe fatigue, which corresponds with a previously reported level of fatigue in the general Norwegian population.

## CONFLICT OF INTEREST

The authors declare no conflicts of interest.

### PEER REVIEW

The peer review history for this article is available at https://publons.com/publon/10.1002/brb3.2608.

## Data Availability

The data that support the findings of this study are available from the corresponding author upon reasonable request.
